# Glycemic Control in Type 1 Diabetes Mellitus and COVID-19: What We Learned From the Lockdown Experience

**DOI:** 10.7759/cureus.33340

**Published:** 2023-01-04

**Authors:** Catarina Almeida, André Ferreira, Daniela Duarte, Ana Filipa Viegas, André Santos, Alexandra Vaz, Edite Nascimento

**Affiliations:** 1 Department of Internal Medicine, Centro Hospitalar Tondela Viseu, Viseu, PRT; 2 Department of Nephrology, Centro Hospitalar Tondela Viseu, Viseu, PRT

**Keywords:** covid-19 lockdown, glycemic control, sars-cov-2, covid-19, type 1 diabetes mellitus (t1d)

## Abstract

Introduction: Confinement measures that were imposed during the COVID-19 pandemic drastically changed the routines of the population. Some studies on the impact of confinement on glycemic control suggest a reduction of 0.1 to 0.5% in glycated hemoglobin. The objective of this study was to evaluate the impact of the COVID-19 pandemic lockdown on glycemic control in adult patients with type 1 diabetes mellitus.

Methods: An observational retrospective cohort study of patients with type 1 diabetes mellitus followed in a Diabetes Unit was performed. The study compared the metabolic control of these patients before (between January 1st and March 18th, 2020) and after (between May 3rd and July 31st, 2020) the lockdown.

Results: The study included 102 patients with type 1 diabetes mellitus (51% females), with a median age of 36 years (interquartile range 18.75, (24.25-43)) and a median duration of diabetes of 15 years (interquartile range 13, (8-21)). After lockdown, a significant decrease of 0.28±0.71% in glycated hemoglobin was observed (7.88±1.33% *vs* 7.59±1.23%, *p*=<0.001). In patients using continuous glucose monitoring a significant improvement in time in range was also noted (47.25±17.33% *vs* 49.97±18.61%, *p*=0.008).

Conclusions: This study demonstrated an improvement in glycemic control after the lockdown. This might be explained by the positive impact of stable schedules, healthy meals and greater availability to make therapeutic adjustments to glycemic control. The fact that diabetes was considered a risk factor for the development of severe COVID-19 disease might also influence patients to increase their efforts to optimize their glycemic control.

## Introduction

In December 2019, a new virus known as severe acute respiratory syndrome coronavirus 2 (SARS-CoV-2) emerged in Wuhan, China [1]. It rapidly spread worldwide and on March 11th, 2020, it was defined as a pandemic disease by the World Health Organization [2]. The most vulnerable people to this virus are those with chronic diseases, such as diabetes mellitus [3,4].

Coronavirus disease 2019 (COVID-19) forced governments and public health authorities, in many countries, to impose exceptional measures to prevent the spread of this disease [5]. Restrictions to circulation, social distancing, school closure and mandatory remote working whenever possible, were some of the adopted measures, which drastically changed people’s personal and professional lives [3,5-7]. In Portugal, a state of emergency was declared on March 18th, 2020, and stringent containment and mitigation measures were imposed, until May 2nd, 2020. During this period, access to health care was compromised and outpatient consultations were postponed or performed remotely.

The impact that changes in daily routines have on glycemic control in patients with type 1 diabetes mellitus (T1D) is well-known. Diet, physical activity, compliance with therapy, as well as work activity, emotional stress and social life are some factors that significantly influence glycemic control [5].

Achieving and maintaining glycemic targets in people with T1D has always been a challenge, which became even more significant during the COVID-19 pandemic when access to health care was restricted and the importance of adequate self-management increased [5]. However, in contrast to what would be expected, the implementation of lockdown during the COVID-19 pandemic did not appear to worsen glycemic control in patients with T1D. Some studies on the impact of confinement on glycemic control suggest a reduction of 0.1 to 0.5% in the value of glycated hemoglobin (HbA1c) [6-11].

The aim of this study was to assess the impact of the lockdown due to COVID-19 on glycemic control in adult patients with T1D followed in our Unit.

## Materials and methods

We performed an observational retrospective cohort study of T1D patients, aged 18 and above, followed in the Diabetes Unit of Centro Hospitalar Tondela-Viseu (CHTV).

The sampling was non-randomized and consecutive, gathering all available subjects. All T1D patients with an outpatient appointment between January 1st and March 18th, 2020 and with reassessment between May 3rd and July 31st, 2020, were eligible. Exclusion criteria included: 1) unknown information about HbA1c and continuous glucose monitoring (CGM), 2) pregnancy during the period of the study, 3) changes in insulin therapy regimen (between multiple daily insulin injections [MDI] and continuous subcutaneous insulin infusion system [CSIS]), in the six months before the study, and 4) diagnosis of T1D in the year prior to the study.

Data collection was made from the hospital's digital database. It included 1) socio-demographic data (gender, age and T1D duration); 2) clinical data regarding T1D status (insulin therapy regimen); and 3) data regarding metabolic control before and after lockdown (body weight, HbA1c and information from CGM). Information from CGM corresponds to the analysis of daily glycemic variation during a 28-day period, in particular, mean glucose and percentage of time in glucose range (TIR) (glucose between 70-180 mg/dL), time below range (TBR) (glucose <70 mg/dL) and time above range (TAR) (glucose >180 mg/dL).

This study was approved by the Ethics Committee of CHTV. All the data were anonymized for processing.

Statistical analysis was performed using Statistical Package for Social Sciences (SPSS) version 28 (IBM Corp., Armonk, NY, USA). For the descriptive analysis, continuous variables were assessed with central distribution (mean and median) and dispersion measures (standard deviation, minimum, maximum and interquartile range [IQR]) and categorical variables were assessed with measures of absolute and relative frequency. For the inferential analysis, means of the normally distributed variables were compared using parametric tests; medians of the non-normally distributed variables were compared using non-parametric tests. A *p* value less than 0.05 was considered statistically significant.

## Results

A cohort of 102 patients with T1D was analyzed. The median age was 36 years old (IQR 18.75, (24.25-43); minimum 18, maximum 79), 51% were female (n=52) and the median duration of T1D was 15 years (IQR 13, (8-21); minimum 1, maximum 45). Seventy-two percent of the patients (n=74) followed an MDI regimen, whereas 28% (n=28) of them used a CSIS. Eighty-five percent of the patients (n=87) used CGM. None of the participants had COVID-19 during the study period.

Table 1 presents the comparison of glycemic metrics before (January 1st to March 18th, 2020) and after (May 3rd to July 31st, 2020) lockdown. A significant decrease of 0.28±0.71% in HbA1c was observed (7.88±1.33% *vs* 7.59±1.23%, *p*=<0.001). In patients using CGM (n=87) a significant improvement of 3.70±11.24% in TIR was also observed (47.25±17.33% *vs* 49.97±18.61%, *p*=0.008). A decrease of 3.70±39.34mg/dL in mean glucose (178.41±37.82mg/dL *vs* 172.86±38.60mg/dL, *p*=0.509), 3.12±16.76% in TAR (46.32±19.50% *vs* 43.21±20.01%, *p*=0.127) and 0.35±12.79% in TBR (6.82±12.06% *vs* 6.11±6.37%, *p*=0.822) were found, but they were not statistically significative.

**Table 1:** Comparison of glycemic metrics before (January 1st to March 18th, 2020) and after (May 3rd to July 31st, 2020) lockdown. CMG – Continuous glucose monitoring, HbA1c - glycated hemoglobin, SD – Standard deviation.

	Before lockdown	After lockdown	p
General population (n=102)			
HbA1c (%, mean ± SD)	7.88 ± 1.33	7.59 ± 1.23	<0.001
Body weight (Kg, mean ± SD)	71.63 ± 13.90	71.40 ± 13.92	0.578
Patients with CGM (n=87)			
Mean glucose (mg/dl, mean ± SD)	178.41 ± 37.82	172.86 ± 38.60	0.509
Time in range, 70–180 mg/dl (%, mean ± SD)	47.25 ± 17.33	49.97 ± 18.61	0.008
Time above range, > 180 mg/dl (%, mean ± SD)	46.32 ± 19.50	43.21 ± 20.01	0.127
Time below range, < 70 mg/dl (%, mean ± SD)	6.82 ± 12.06	6.11 ± 6.37	0.822

There was no statistically significant difference in the body weight (71.63±13.90Kg *vs* 71.40±13.92Kg, *p*=0.578) between the two periods.

A reduction of 0.5% or more in HbA1c was found in 35.3% (n=36) of the subjects. In patients with CGM, 42.5% (n=37) had an increase of 5% in TIR.

Comparing patients with (n=87) and without (n=15) CGM, a decrease in HbA1c was observed in both groups (7.79±1.17 *vs* 7.54±1.16, *p*=0.001 and 8.43±1.99 *vs* 7.92±1.59, *p*=0.056, respectively), but it was only statistically significant in patients with CGM.

Regarding insulin therapy regimen (Table 2), it was noted a statistically significant reduction of 0.29±0.71% in HbA1c (8.07±1.42% *vs* 7.78±1.28%, *p*=<0.001), in patients with MDI (n=74). In patients with MDI using CGM (n=60) a significant improvement of 3.76±11.41% in TIR was also observed (43.54±17.04% *vs* 46.49±17.78%, *p*=0.033). In patients with CSIS (n=28) a decrease of 0.26±0.73% in HbA1c was observed (7.36±0.87% *vs* 7.10±0.95%, *p*=0.085), but it was not statistically significant. In patients with CSIS using CGM (n=27) an improvement of 3.60±11.16% in TIR was also observed (54.11±15.96% *vs* 57.08±18.58%, *p*=0.120). Information about mean glucose, TAR and TBR is described in Table 2.

**Table 2:** Comparison of glycemic metrics before (January 1st to March 18th, 2020) and after (May 3rd to July 31st, 2020) lockdown, according to insulin regimen (multiple daily insulin injections vs continuous subcutaneous insulin infusion system). CMG – Continuous glucose monitoring, CSIS - continuous subcutaneous insulin infusion system, HbA1c - glycated hemoglobin, MDI - multiple daily insulin injections, SD – Standard deviation.

	Before lockdown	After lockdown	p
Patients with MDI regimen (n=74)			
HbA1c (%, mean ± SD)	8.07 ± 1.42	7.78 ± 1.28	<0.001
Patients with MDI regimen and CGM (n=60)			
Mean glucose (mg/dl, mean ± SD)	186.94 ± 41.94	176.12 ± 40.40	0.572
Time in range, 70–180 mg/dl (%, mean ± SD)	43.54 ± 17.04	46.49 ± 17.78	0.033
Time above range, > 180 mg/dl (%, mean ± SD)	48.82 ± 19.67	45.96 ± 18.40	0.276
Time below range, < 70 mg/dl (%, mean ± SD)	7.54 ± 14.38	6.02 ± 6.29	0.648
Patients with CSIS (n=28)			
HbA1c (%, mean ± SD)	7.36 ± 0.87	7.10 ± 0.95	0.081
Patients with CSIS and CGM (n=27)			
Mean glucose (mg/dl, mean ± SD)	166.80 ± 28.19	164.70 ± 33.23	0.666
Time in range, 70–180 mg/dl (%, mean ± SD)	54.11 ± 15.96	57.08 ± 18.58	0.120
Time above range, > 180 mg/dl (%, mean ± SD)	41.50 ± 18.58	37.60 ± 22.30	0.259
Time below range, < 70 mg/dl (%, mean ± SD)	5.42 ± 5.29	6.28 ± 6.65	0.439

In metabolic control analysis (Table 3), a statistically significant decrease of 0.43±0.74% in HbA1c (8.67±1.08% *vs* 8.25±1.03%, *p*=<0.001) was observed in patients with worst metabolic control (defined as HbA1c >7.5%; n=61). In patients with worst glycemic control using CGM (n=51) an increase of 2.84±11.47% in TIR (37.39±14.06% *vs* 40.48±16.41%, *p*=0.135) was observed but it was not statistically significant. In patients with better metabolic control (defined as HbA1c ≤7.5%; n=41) an improvement of 0.07±0.61% in HbA1c was observed (6.68±0.54% *vs* 6.61±0.77%, *p*=0.490), but it was not statistically significant. Patients with better glycemic control using CGM (n=36) had, however, a significant improvement of 4.48±11.16% in TIR (58.06±13.47% *vs* 62.32±12.20%, *p*=0.033) and a significant decrease of 4.97±12.21% in TAR (34.59±15.51% *vs* 29.45±14.85%, *p*=0.034). Further information about glycemic metrics is presented in Table 3.

**Table 3:** Comparison of glycemic metrics before (January 1st to March 18th, 2020) and after (May 3rd to July 31st, 2020) lockdown, according to metabolic control. CMG – Continuous glucose monitoring, HbA1c - glycated hemoglobin, SD – Standard deviation.

	Before lockdown	After lockdown	p
Patients with worst metabolic control, HbA1c >7.5% (n=61)			
HbA1c (%, mean ± SD)	8.67 ± 1.08	8.25 ± 1.03	<0.001
Patients with worst metabolic control, HbA1c >7.5%, and CGM (n=51)			
Mean glucose (mg/dl, mean ± SD)	196.00 ± 38.68	188.85 ± 37.89	0.767
Time in range, 70–180 mg/dl (%, mean ± SD)	37.39 ± 14.06	40.48 ± 16.41	0.135
Time above range, > 180 mg/dl (%, mean ± SD)	56.56 ± 16.68	53.57 ± 16.69	0.655
Time below range, < 70 mg/dl (%, mean ± SD)	5.90 ± 15.15	5.23 ± 5.58	0.869
Patients with better metabolic control, HbA1c ≤7.5% (n=41)			
HbA1c (%, mean ± SD)	6.68 ± 0.54	6.61 ± 0.77	0.490
Patients with better metabolic control, HbA1c ≤7.5%, and CGM (n=36)			
Mean glucose (mg/dl, mean ± SD)	157.69 ± 24.42	150.54 ± 27.22	0.286
Time in range, 70–180 mg/dl (%, mean ± SD)	58.06 ± 13.47	62.32 ± 12.20	0.033
Time above range, > 180 mg/dl (%, mean ± SD)	34.59 ± 15.51	29.45 ± 14.85	0.034
Time below range, < 70 mg/dl (%, mean ± SD)	8.06 ± 7.01	7,48 ± 7.24	0.850

## Discussion

Opposite to what would be expected during a period with difficult access to health services, increased levels of anxiety and reduced physical activity [5,12], we found an improvement in glycemic control in our sample. We documented a significant decrease of 0.28% in HbA1c after the lockdown period which is consistent with other international studies [5-11,13,14]. We further determined a relevant reduction in HbA1c (≥0.5%) in more than one-third (35.3%) of our population. An increase of TIR of 3.70% after the COVID-19 lockdown was also observed in a similar percentage to other studies [5,6,9-11,13-16]. This increase in TIR was accompanied by a decrease in TAR and TBR, although not statistically significant. Only the subgroup of patients with CSIS had a slight increase in TBR of 0.86% which was not statistically significant.

The COVID-19 pandemic represented a global health crisis, transforming the way how non-critical patients had access to healthcare systems. In many centers, outpatient appointments were performed remotely, in a system of telemedicine, to reduce the risk of exposure of vulnerable patients. In our unit, all scheduled appointments were postponed, but diabetic patients could contact our team, by phone call, to clarify any doubts or to request a medical evaluation. We considered that this support was essential to obtain these good results and many studies suggest the importance of telemedicine in glycemic control during the lockdown period [7,13,17].

CGM is one of the most important technological innovations in the field of diabetes and became essential in the management of these patients [18]. CGM provides real-time information about glucose levels, as well as, glucose trends and rate of change; this assists patients to make therapeutic adjustments, so they can self-manage their diabetes more efficiently and improve metabolic control [6,19,20]. In our sample, we found a decrease in HbA1c in both patients with and without CGM, but it was only statistically significant in patients with CGM. We only had 15 patients without CGM, so it is difficult to take any conclusions, however, we believe that these devices contributed to the improvement of glycemic control during lockdown. The possibility of sharing information from CGM virtually has also become an essential part of telemedicine [6].

Although it is difficult to assess the underlying reasons for this improvement in glycemic control, we also hypothesized that a predictable daily routine, with time to focus on diabetes self-care, in the absence of the usual daily stress, could have overcome the negative impact that the pandemic had in diabetic patients. They had more time to make frequent assessments of glucose levels, performed therapeutic adjustments and count carbohydrates, which was facilitated by the necessity to prepare meals at home. This might explain the results seen in our study since many of our patients were working or studying from home. This theory was also supported by other authors [5-7,10,14,16].

Concerning body weight variations, studies are not consistent, with some studies reporting deterioration in body weight, whereas others showed stability or improvement in body weight [3,6,13]. Our patients maintained relative stability of body weight. This suggests that they have a high level of awareness and could balance food intake and home exercise to keep better control of their glycemic pattern.

Our analysis showed that both patients with MDI and CSIS had a decrease in HbA1c (0.29% and 0.26%, respectively) and an increase in TIR (3.76% and 3.60%, respectively); however, these improvements were only statistically significative in patients with MDI regimen. These results might be explained by the fact that patients with CSIS had already better metabolic control before the lockdown and reinforced their attitudes to keep good control.

On the other hand, we have to emphasize that patients with the worst glycemic control (HbA1c >7.5%) had a greater improvement in HbA1c compared to patients with better glycemic control (HbA1c ≤7.5%) (0.43% and 0.07%, respectively). This could be attributed to the fact that diabetes was widely reported as a risk factor for developing severe COVID-19 disease [6,11,14]. Good metabolic control might help reduce the severity of the disease [13]; considering this premise, T1D patients might increase their efforts to optimize their glycemic control and potentially mitigate this risk.

Some potential limitations of our study should be noticed. It is a single-center, retrospective observational study and has a small sample, which decreases its statistical power. Without data from a control group, it is not possible to infer causality, and we can only describe an association between lockdown and improvements in glycemic metrics. We were unable to collect data on glucose management indicator and glucose variability. The lack of information about changes in exercise and dietary habits, as well as changes in working routine, is also a limitation.

Further research to determine the reasons for glycemic control improvement is needed so people with diabetes can continue to benefit from the lessons learned during the COVID-19 pandemic.

## Conclusions

Our study demonstrates that glycemic metrics in patients with T1D significantly improved after the COVID-19 lockdown. These results highlight the importance of a stable daily routine in patients with T1D, although we can not exclude that other factors might also influence patients to optimize their glycemic control.

It is also important to highlight that diabetes technology and telemedicine, overcome physical limits and can improve healthcare access so they are important tools to develop to provide better care to T1D patients.
